# A Deep-Learning Framework for the Detection of Oil Spills from SAR Data

**DOI:** 10.3390/s21072351

**Published:** 2021-03-28

**Authors:** Mohamed Shaban, Reem Salim, Hadil Abu Khalifeh, Adel Khelifi, Ahmed Shalaby, Shady El-Mashad, Ali Mahmoud, Mohammed Ghazal, Ayman El-Baz

**Affiliations:** 1 Electrical and Computer Engineering, University of South Alabama, Mobile, AL 36688, USA; mshaban@southalabama.edu; 2 College of Engineering, Abu Dhabi University, Abu Dhabi 59911, United Arab Emirates; reem.salim@adu.ac.ae (R.S.); hadil.abukhalifeh@adu.ac.ae (H.A.K.); adel.khelifi@adu.ac.ae (A.K.); mohammed.ghazal@adu.ac.ae (M.G.); 3 Bioengineering Department, University of Louisville, Louisville, KY 40292, USA; ahmed.shalaby@louisville.edu (A.S.); ahmahm01@louisville.edu (A.M.); 4 Faculty of Engineering, Benha University, Benha 13511, Egypt; shady.elmashad@feng.bu.edu.eg

**Keywords:** Synthetic Aperture Radar (SAR), oil spill, deep learning

## Abstract

Oil leaks onto water surfaces from big tankers, ships, and pipeline cracks cause considerable damage and harm to the marine environment. Synthetic Aperture Radar (SAR) images provide an approximate representation for target scenes, including sea and land surfaces, ships, oil spills, and look-alikes. Detection and segmentation of oil spills from SAR images are crucial to aid in leak cleanups and protecting the environment. This paper introduces a two-stage deep-learning framework for the identification of oil spill occurrences based on a highly unbalanced dataset. The first stage classifies patches based on the percentage of oil spill pixels using a novel 23-layer Convolutional Neural Network. In contrast, the second stage performs semantic segmentation using a five-stage U-Net structure. The generalized Dice loss is minimized to account for the reduced oil spill representation in the patches. The results of this study are very promising and provide a comparable improved precision and Dice score compared to related work.

## 1. Introduction

An oil spill is an environmental threat that has adverse effects on bodies of water, land, and air [1]. Further, it can cause pollution to sea surfaces and harm bird species, fish, and other aquatic creatures. They are primarily caused by accidents involving oil tankers, ships, and pipelines where crude oil, gasoline, fuel, and oil by-products are released into the water. Removal of oil slicks is crucial to maintain a safe and clean environment and protect aquatic life.

Synthetic Aperture Radar (SAR) is usually mounted on aircraft or satellites to obtain images for sea and land surfaces [1]. Sensors deployed by the SAR send radio waves that are then reflected off the surfaces, allowing for a visual representation of the target surface. Captured SAR images may include sea, land surfaces, oil spills, ships, and look-alikes. Look-alikes may represent a vast range of environmental phenomena, including low-speed wind areas, sea wave shadows, and grease ice. Radio waves reflected by oil spills or look-alikes are represented as dark or black spots in SAR images, identifying oil spills and discrimination from other look-alikes a challenge.

Several approaches were recently investigated to classify and segment oil spills using deep learning and neural networks classifiers [1,2,3,4,5,6,7,8,9,10,11,12,13,14,15,16,17,18,19,20,21,22,23,24,25,26,27,28]. Although most of these approaches are promising, they rely on handcrafted feature extraction (i.e., semi-automatic classification) or the classification of oil spills with modest accuracy or a Jaccard score.

In this study, we introduce a two-stage convolutional network to classify and segment images with oil spills. The first stage is realized using a novel 23-layer Convolutional Neural Network (CNN) that can classify patches into less than 1% oil spill patches and more than 1% oil spill patches with a five-fold well as ten-fold accuracy, sensitivity, and specificity of almost 99%. The second stage takes the patches with significant oil spill presences. Oil spill instances constitute more than 1% of the entire patch resolution and segment them using a five-stage U-Net with an optimized generalized Dice loss. The proposed framework accurately detected oil spill pixels with an accuracy of 92%, a precision of 84%, and a Dice score of 80%. The proposed study provides improved precision and Dice score compared to a state-of-the-art architecture.

The remainder of this paper is organized as follows. Section 2 provides a review of the related work followed by a description of the used dataset and proposed approach in Section 3. The experimental study and results are discussed in Section 4. Finally, the conclusion and summary are presented in Section 5.

## 2. Related Work

Several works have adopted semantic segmentation and deep convolutional neural networks to detect the oil slicks on the sea surface [1,2,3,4,5,6,7,8,9,10,11,12,13,14,15,16,17,18,19,20,21,22,23,24,25,26,27,28]. Del Frate et al. introduced a multilayer perceptron with 11-8-8-1 topology that classifies instances into oil spills and look-alikes based on a features vector extracted from the dataset after specifying the area of interest is usually a dark spot [1]. An overall of 18% of oil spill instances and 10% of look-alikes were misclassified. De Souza et al. introduced a similar approach with an area of interest specified by a human operator [2]. Stathakis et al. introduced a feature selection approach based on a genetic algorithm that searches for the best set of features and the number of activation maps generated by the hidden layers of a neural network classifier [3]. The proposed approach provided a classification accuracy of 84.8% with an improvement of 7.6% over the standard approach, which uses all features as inputs for the neural network classifier.

Topouzelis et al. introduced two artificial neural networks for oil spill detection [4]. The first network with 1-3-1 topology detects dark formation with an accuracy of 94%. Further, a set of ten features were extracted from the dark formation, and a fully connected multilayer perceptron with 10-51-2 topology was applied on the features obtained, providing an accuracy of 89% for discriminating oil spills from look-alikes. Singha et al. also proposed two neural networks in a sequence to detect oil spills [5]. The first network with 3-6-2 topology is used to segment the SAR images to a dark formation or background, while a second neural network with 14-14-5-1 topology is used to identify a pixel as an oil spill or look-alike. The proposed network correctly classified 91.6% of oil spill pixels and 98.3% of look-alike pixels.

Song et al. applied an optimized wavelet neural network on a selected set of fully polarimetric features extracted from SAR images [6] with an overall accuracy of 96.55% and 97.67% on two different datasets. Chen et al. introduced Stacked Autoencoder (SAE) and deep belief networks on polarimetric SAR features to detect oil spill instances [7]. The use of the aforementioned deep-learning approaches was shown to be promising as compared to Support Vector Machine (SVM) and typical artificial neural networks with a testing error below 1.4% when the SAE was used. Gallego et al. introduced the use of deep autoencoders to address the oil spill segmentation problem [8,13]. The authors used two deep autoencoders with 4 and 6 layers, respectively, on Side-Looking Airborne Radar images [8]. Five-fold cross-validation was used, with 80% of the samples used for training and 20% used for testing. Authors claimed that one of the deep autoencoders achieved a Jaccard score of 1 and *f*-1 score of 0.93, while the other achieved a Jaccard score of 0.92 and *f*-1 score of 0.89.

Orfanidis et al. introduced the use of DeepLab model with ResNet 101 as a base semantic segmentation network [9] for pixel-wise classification into the oil spill, look-alike, and background. The authors applied localization, cropping, radiometric calibration, speckle filtering, and linear transformation from dB to actual luminosity values prior to segmentation. The model introduced a Jaccard score of 0.41 for oil spills. A patch image segmentation was introduced where patches were picked under certain constraints elevating the Jaccard score for oil spill patches to 0.86 for 3 × 3 patches and 0.89 for 5 × 3 patches. Hidalgo et al. introduced a two-stage CNN for coarse detection of ships, oil spills, and coasts and precise detection at the pixel level [10] with the highest accuracy, precision, recall, and *f*-1 score of 99%, 65%, 86.8%, and 71%, respectively.

Yu et al. introduced a deep neural network that minimizes the *f*-divergence between the ground truth and predicted segmentation masks [11]. For the two oil spill region cases, the proposed approach provided a 15% accuracy increase and a 25% Region Fitting Error (RFE) decrease for the Gamma noise concerning the Generative Adversarial Network (GAN). Further, for the three oil spill region cases, the proposed method outperformed GAN in terms of RFE, with decreases in the range of 25.5% to 33% for Gamma noise, the range of 46.5% to 50.7% for Rayleigh noise, and the range of 49% to 51% for log-normal noise.

Liping et al. investigated and provided long-term prediction for the drifting path of the oil-contaminated water due to a collision of the Sanchi oil tanker in the East China Sea using the oceanic surface wave-tide-circulation coupled forecasting system (OFS) developed by the First Institute of Oceanography, State Oceanic Administration (SOA), China [12]. Further, Qia et al. proposed a 3D model for short-term and long-term oil spill paths caused by the Sanchi tanker [19]. Guo et al. introduced the use of SegNet to segment oil spills represented as dark spots in SAR images [14] with an accuracy of 93% under high noise. Further, Li et al. introduced the use of polarimetric SAR filters (e.g., Boxcar, Refined Lee, and Lopez filters) to extract respective polarimetric SAR features and feed the features to a stacked autoencoder [15]. Authors were able to distinguish between crude oil, biogenic slicks, and clean seawater using the Lopez filter and autoencoder with an overall accuracy of 99.38%.

Jiao et al. introduced a pre-trained deep convolutional neural network based on VGG-16 for classifying oil spill instances and the Otsu algorithm to reduce the false positive rate and Maximally Stable Extremal Regions (MSER) algorithm for locating the oil spill by generating a detection box [16]. The VGG-16 achieved a recall of 99.5% and an *f*-measure of 98.5%. The use of the Otsu algorithm improved the precision from 97.7% to 98.3%, while the oil spill was marked using the MSER method at a proper threshold setting. Zhu et al. experimented with SVM, fully connected neural networks, SAE, and CNN on hyperspectral remote sensing images for oil film thickness classification [17]. The CNN provided better accuracy and performance as compared with SAE by almost 5% improvement. Krestenitis et al. generated a pre-processed SAR images dataset acquired by the Sentinel-1 satellites of the European Space Agency (ESA) [18]. Further, UNet, LinkNet, PSPNet, DeepLabV2, and DeepLabV3+ with MobileNetV2 as a base network were trained on the dataset and provided modest Jaccard scores for the oil spill (i.e., 0.54, 0.52, 0.4, 0.26, and 0.53 respectively).

Yang et al. proposed a seven-layer CNN applied on features extracted at various scales using the Wavelet transform of ALSA+ hyperspectral remote sensing images [20]. The proposed approach achieved an accuracy above 85%. Park et al. introduced the use of artificial neural networks to detect oil leaks in optical PlanetScope satellite images acquired close to Ras Al Zour town in Kuwait [21]. Sun glint effects and dust were subtracted from the images and then provided to an artificial neural network to classify the target pixels into three types of oil leaks and sea surfaces with an overall accuracy of 82%. Li et al. introduced a one-dimensional CNN that classifies the oil film based on the detection of the associated spectral bands with an overall accuracy of 83% [22]. Zeng et al. introduced an oil spill CNN for oil spill detection on Spaceborne SAR images [23]. The proposed approach is based on VGG-16 applied on dark patches obtained by a dark patch generation algorithm from SAR images. The proposed network achieved an accuracy of 94%, a recall of 83.5%, a precision of 85.7%, and an *f*-measure of 84.6%.

Yekeen et al. [24,25] introduced the use of mask-region-based CNN to distinguish between ships, oil spills, and look-alikes where pre-trained ResNet 101 and feature pyramid network were used for feature extraction, regional proposal network was deployed for the region of interest extraction, and the mask-region-based CNN was used for semantic segmentation. The proposed model introduced a classification accuracy of 96%, and 92% for oil spills and look-alikes, respectively. Bianchi et al. proposed an oil-based fully convolutional network where the weighted cross-entropy loss is minimized in order to segment, detect, and categorize oil spills into one of 12 categories [26]. Zhang et al. introduced a semantic segmentation approach based on CNN, which receives different polarized parameters generated via four channels processed by Lee Refined filter and Simple Linear Iterative Clustering (SLIC) superpixel [27]. The proposed approach attained a mean intersection of 90.5% when the Yamaguchi parameters were extracted as feature sets. Further, Baek et al. addressed the problem of detecting oil spills dual-polarized Terra-SAR-X images using artificial and convolutional neural network regression models with *f1*-score of 0.83 and 0.82, and AUC of 0.986 and 0.987, respectively [28].

## 3. Materials and Methods

### 3.1. Dataset

The dataset used in this study was acquired by the Sentinel-1 satellites and distributed by European Space Agency (ESA) via the Copernicus Open Access Hub [29]. The dataset consists of 310 images pre-processed via cropping sequence, radiometric calibration, filtering to reduce speckle noise, and a linear transformation to convert dB to real luminosity values [30]. A resolution of 1250 × 650 × 3 pixels was maintained. Dataset images include instances for oil spills, look-alikes, ships, land, and sea. Both oil spill and look-alike instances are depicted as elongated dark and large black areas, respectively. Three-dimensional labels were generated for images where cyan, red, brown, green, and black masks represent the oil spill, look-alike, ships, land, and sea, respectively.

**Figure 1:**
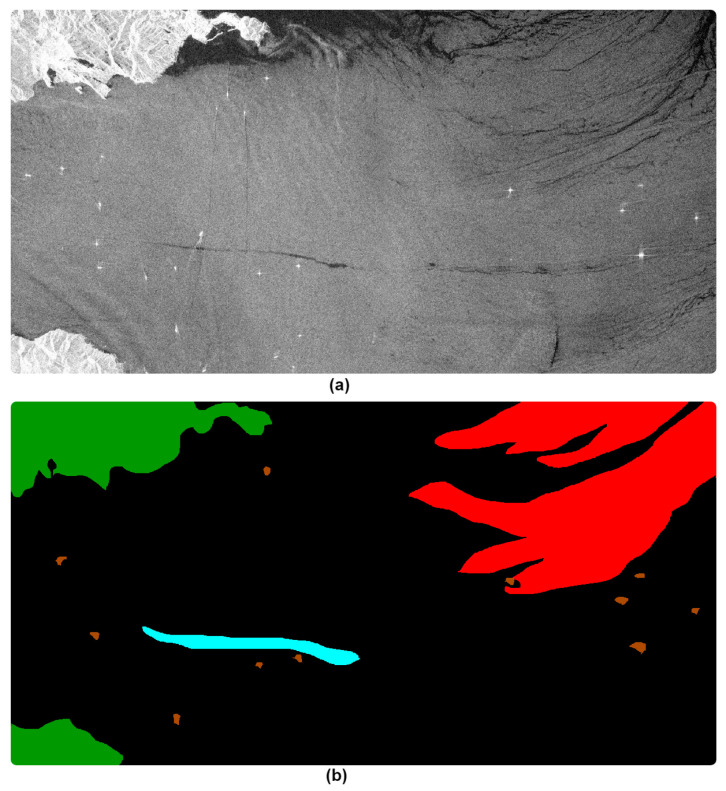
(**a**) SAR image depicting sea, oil spill, look-alikes, ships, and land; (**b**) corresponding three-dimensional masks.

Figure 1 shows an example of a SAR image and the corresponding 3-D mask. Further, one-dimensional labels were provided for further classification and segmentation where 0, 1, 2, 3, 4, and 5 were assigned to the sea surface, oil spill, look-alike, ship, and land, respectively. Table 1 shows a summary of the one- and three-dimensional label assignments as well as the pixel counts. Table 1 shows that Oil Spill instances only represent 1.2% of the total pixels, creating a hurdle for a direct semantic segmentation using fully convolutional neural network models as the dataset suffers severe imbalance. Further, in this study, we have discarded images that containing neither oil spills nor look-alike instances during the training and learning process, which increases the imbalance of the dataset. A total of 210 images were then used in this study. Also, assigned pixels belonging to sea, land, ship, and look-alike instance labeled “0” representing the background, whereas oil spill pixels were labeled as “1”.

### 3.2. Deep-Learning Framework

**Figure 2:**
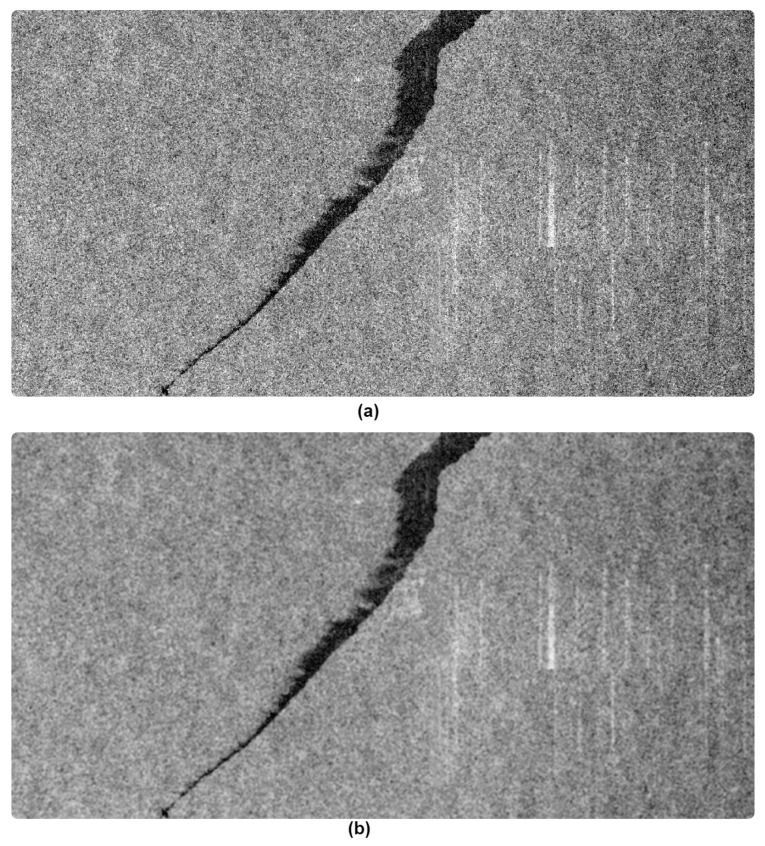
(**a**) Before applying the Frost filter. (**b**) After applying the Frost filter.

The proposed framework, as shown in Figure 2, starts with applying a frost filter on SAR images to reduce the Speckle noise present in the background while preserving the edges of the oil spill regions. The coefficient of the matrix that represents the Frost filter kernel at the pixel location (*x, y*) is defined as follows [31]:

(1) $$W(x,y)=e^{-\frac{K\sigma^{2}r}{\mu^{2}}}$$

where *x,* and *y* refer to the row and the column of the coefficient within the matrix respectively, *μ* and *σ* are the mean and the standard deviation of the corresponding neighborhood values of the image where the Frost filter kernel is applied, and *K* is the rate at which the coefficients of the matrix decay away from the matrix center and origin. Further, *r* is the Euclidean distance from the center pixel of the kernel to the pixel located at (*x*,*y*). Figure 2 shows a SAR image before and post-application of the Frost filter. The speckle noise was reduced in the background without affecting the dark object (i.e., oil spill). The reduction also reduces the number of dark pixels in the background that can be confused with oil spill instances.

**Figure 3:**
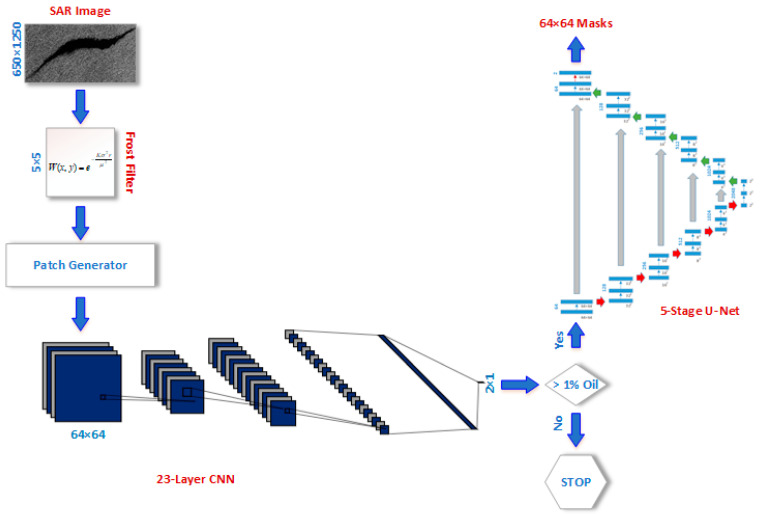
Proposed deep-learning framework.

Then, the denoised images were split into patches of size 64 × 64 × 3. Further, patches are split into two groups; patches with greater than 1% oil pixels and patches with lower than 1% oil spill pixels. Patches with 1% oil spill have almost 40 pixels out of an overall 4096 pixels with a “1” label that can safely be considered background patches. Further, the patches with less than 1% oil spill increase the imbalance of the dataset and reduce the model’s accuracy if they are considered with other patches. Patches are then provided to a two-stage deep-learning framework, as shown in Figure 3. The first stage uses a novel CNN that consists of 23 convolutional, rectified linear units (ReLU), pooling, fully connected, and SoftMax layers and defined in Table 2. At the SoftMax layer, the cross-entropy (i.e., predicted probability distribution of each output generated by each node of the final two-node fully connected layer) is calculated, and defined as follows:

(2) $$\tilde{P}(c_{i})=\frac{e^{c_{i}}}{\sum_{j=1}^{2}e^{c_{j}}}$$

where *c_i_* represents the output of the node *i* of the fully connected layer and $\tilde{P}(c_{i})$ is the predicted cross-entropy of *c_i_*. Finally, the cross-entropy for each of the two outputs is compared with respect to some threshold, and a decision regarding the input patch is predicted (i.e., significant or insignificant oil spill patch). The cross-entropy loss (CEL) is also calculated and minimized via a backpropagation gradient descent algorithm in order to update the parameters of the CNN model and offer an accurate prediction for the patch class. *CEL* is defined as follows:

(3) $$CEL=-\sum_{i=1}^{2}P(c_{i})\log[\tilde{P}(c_{i})]$$

where *P(c_i_)* is the desired or expected probability distribution of the output value *c_i_*.

**Figure 4:**
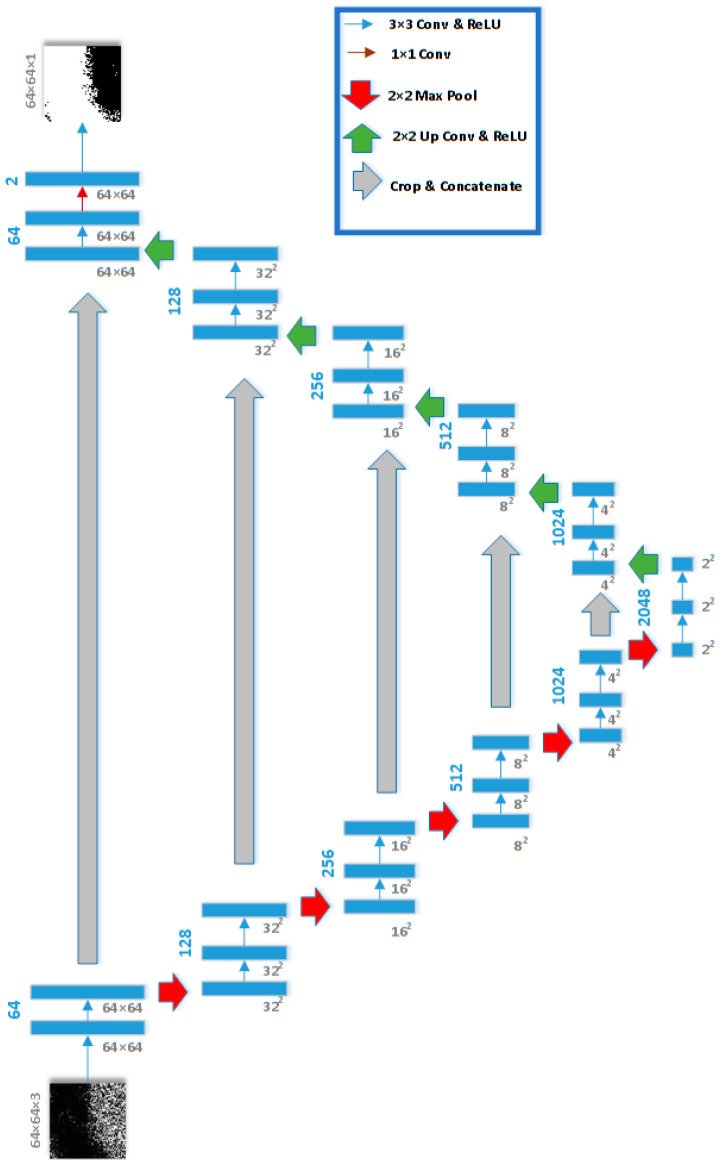
Five-stage U-Net Architecture for the Semantic Segmentation of Oil Spills.

The second stage of the proposed framework, shown in Figure 4, deploys a five-stage U-Net [32] to segment and detect the oil spill pixels within the correctly classified patches with more than 1% oil spill instances. U-Net architecture is developed where the generalized Dice loss is minimized. Generalized Dice loss (GDL) was adopted in this study to account for the imbalance that is still present in the SAR images. GDL is defined as follows [33]:

(4) $$GDL=1-2\frac{\sum_{l=1}^{2}w_{l}\sum_{n=1}^{4096}r_{\ln}p_{\ln}}{\sum_{l=1}^{2}w_{l}\sum_{n=1}^{4096}r_{\ln}+p_{\ln}}$$

where *r_ln_*, and *p_ln_* are the correct and predicted label for an oil spill voxel *n* within a 64 × 64 × 3 belonging to a class *l*, and *w_l_* is defined as follows:

(5) $$w_{l}=\frac{1}{{\left(\sum_{n=1}^{4096}r_{\ln}\right)}^{2}}$$

The U-Net consists of a five-stage contracting path, a five-stage expansive path, and a bottleneck bridge [30]. Each contracting stage comprises two 3 × 3 unpadded convolutional/ReLU layers (blue arrows) and a single 2 × 2 max pooling layer with a stride of 2 (red arrows). The number of channels in the five stages of the contracting path is 64, 128, 256, 512, and 1024. Every stage of the expansive path consists of a 2 × 2 upsampling, two 3 × 3 convolutional/ReLU layers (blue arrows), and concatenation with a cropped feature map of the corresponding expansive path (gray arrows). A final 1 × 1 convolutional layer is used to generate the corresponding masks of 64 × 64 × 1 pixels.

### 3.3. Evaluation Methods

This study measured the five-fold and ten-fold cross-validation accuracy, sensitivity, specificity, weighted kappa score, and area under the curve (AUC) for the first classification stage.

(6) $$Accuracy\;=\frac{TP+TN}{TP+TN+FP+FN}$$

(7) $$Recall/Sensitivity\;=\;\frac{TP}{TP+FN}$$

(8) $$Specificity\;=\frac{TN}{TN+FP}$$

*TP*, *FP*, *TN*, and *FN* are the true positive, false positive, true negative, and false negative image counts for patch classification into patches with more than or less than 1% oil spill pixels. Area Under the Curve (AUC) for the Receiver Operating Characteristic Curve (ROC) was also provided to determine the classifier’s ability to distinguish between patches with almost no oil spill pixels and patches with oil spill presence. Quadratic weighted Kappa score is another approach that determines the performance and the accuracy of the classifier considering any agreements between predicted and expected labels by chance. This measure provides insights into any bias for the model towards a specific class. The quadratic weighted Kappa score can then be calculated in five steps as follows:

1. Obtain the normalized confusion matrix (*C*).
2. Generate a weights matrix *W* such that the weight value at the location (*i, j*) is defined as follows:
  (9) w(i,j)=(i−j)
3. Create a normalized histogram of both actual labels and predicted label vectors.
4. Calculate the normalized outer product (*P*) of the two histograms.
5. Calculate the quadratic weighted Kappa (*K*) as follows:
  (10) $$K=1-\frac{\sum_{i=0}^{L}\sum_{j=0}^{L}w(i,j)c(i,j)}{\sum_{i=0}^{L}\sum_{j=0}^{L}w(i,j)p(i,j)}$$

In the second stage, we have measured the pixel-wise accuracy, recall, precision, Dice score (i.e., *f*-1 score) for the oil spill instances within each patch. Precision and Dice scores are defined as follows:

(11) $$Precision\;=\frac{TP}{TP+FP}$$

(12) $$Dice\;=\frac{2TP}{2TP+FP+FN}$$

where *TP, FP, TN,* and *FN* are the true positive, false positive, true negative, and false negative pixel counts for the pixel-wise classification.

## 4. Results

A total of 210 images with a resolution of 1250 × 650 × 3 were split into patches of 64 × 64 × 3 by cropping and scanning the images using a kernel with a dimension of 64 × 64 × 3 and a stride of 1. Further, the generated patches are screened for patches with less than 1% oil spill pixels and patches with more than 1% oil spill. We thus allow for the identification of patches with significant oil spill distribution. A total of 199,990 patches were selected, with half the patches having less than 1% oil spill and the other half with more than 1% oil spill. The proposed novel 23-Layer CNN model, as described in Table 1, was trained and validated on the patched dataset via a five-fold and ten-fold cross-validation. Model hyperparameters were selected based upon continuous monitoring for the training loss and training accuracy to avoid model overfitting. The maximum number of training epochs is 40, the learning rate used is 0.00005, and the batch size is 500 patches. Table 3 shows the accuracy, weighted Kappa score, sensitivity, specificity, and AUC for a five-fold and ten-fold cross-validation model. Both the mean and standard deviation of the performance mentioned above measures were calculated.

Based on Table 3, it is evident that the proposed CNN model possesses a superior performance with a validation accuracy, sensitivity, specificity, and a weighted kappa score of 99%. We thus ensure that all patches with high oil distributions are detected to be further segmented using the next stage U-Net.

Patches with more than 1% oil spill are provided to the five-stage U-Net as described in Figure 4 to provide a segmentation mask. To train the U-Net and avoid pixel-wise classification bias towards the most popular class (i.e., background), we have created patches of 64 × 64 × 3 with 40–60% oil spill pixels to maintain balanced pixel-wise classification. As a result, 215,394 patches were extracted from 168 images (80% of the dataset) to be deployed in the U-Net model training. In comparison, testing is done on the 684 patches of the remaining 42 images (i.e., 20% of the dataset). There is no overlap between both the training and testing datasets. Table 4 and Table 5 show the results of the semantic segmentation of the training and testing patches, respectively.

Based on Table 5, the proposed framework produced segmenting patches of 64 × 64 × 3 with an accuracy of 92%, precision of 84%, and Dice score of 80%. By comparing both Table 4 and Table 5, the testing performance results are quite close to the training performance measures indicating a robust generalized model with minimum overfitting. We have also compared the proposed framework with semantic segmentation models directly applied on the dataset where five-stage U-Net, and SegNet [34] with VGG-19 encoder were trained on patches with 40–60% oil spill pixel concentrations with GDL minimized. Table 6 presents the performance measures of the proposed framework and the aforementioned semantic segmentation methods.

As shown in Table 6, although the accuracy and recall of the U-Net, and SegNet were improved as compared with the proposed framework, which is mainly due to the use of GDL loss minimization to update the model parameters, the false positive rate was high, leading to a low precision as well as Dice loss. In addition, we have varied the patch size (i.e., Patch size of 32 × 32, and Patch size of 128 × 128 instead of 64 × 64), and the loss function (i.e., Recall loss, and Jaccard loss [32] where the model sensitivity loss and Intersection over union loss respectively were minimized instead of minimizing the GDL)) as shown in Table 7.

It is obvious from Table 7 that GDL minimization provides a slight improvement over recall loss optimization in terms of precision and Dice scores. However, it provides the same performance as when the Jaccard score is minimized. This is due to the fact that both Jaccard and Dice scores are related. Also, from Table 7, the patch size of 64 × 64 pixels provided by the proposed patch generator is considered an optimum size offering higher precision and Dice score as compared with patches with 32 × 32, and 128 × 128 pixels. It is also observed that as the patch size decreases, the sensitivity of the model is enhanced. This can be well explained due to the less deviation between the number of pixels representing the two different classes when smaller patches are used.

Furthermore, the proposed model offers significantly better precision and Dice score than the two-stage convolutional neural network introduced by Hidalgo et al. [10]. It also generated more accurate predictions rather than a mere use of a segmentation network to predict the oil spill mask in a severely imbalanced dataset as studied by Krestenitis et al. [18]. The proposed model’s performance is comparable to that of Zeng et al. [23] in both precision and Dice score. Table 8 highlights the difference between the proposed framework and work introduced in [10,23].

**Figure 5:**
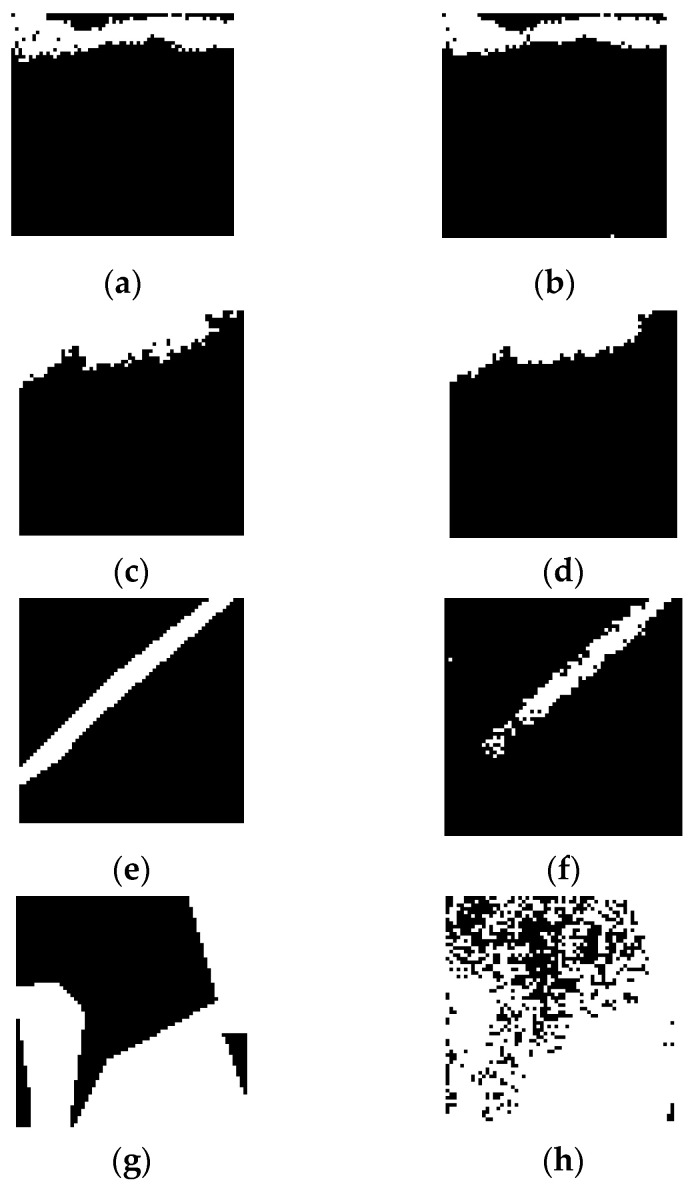
Testing data ground truth and predicted masks such that (**a**,**c**,**e**,**g**) ground truth labels; (**b**,**d**) predicted masks with more than 90% accuracy; (**f**) predicted mask with more than 70% accuracy; (**h**) predicted mask with slightly over 50% accuracy.

Figure 5 shows examples of predicted labels using the proposed deep learning framework under different class imbalance conditions and their corresponding ground truth masks. As shown in Figure 5, the proposed model was able to detect oil spills in patches with relatively balanced pixel labels. Simultaneously, it could not accurately segment patches with relatively higher background distribution as in Figure 5e or relatively higher oil spill distribution as shown in Figure 5g.

## 5. Discussion

In this study, we have addressed the challenge of detecting and segmentation irregular-sized oil spill instances that constitute a small portion of low-resolution spaceborne SAR images using deep-learning structures. Semantic segmentation using fully convolutional, U-Net, and other segmentation architecture has failed to accurately detect the spills described by Krestenitis et al. [18], where the highest Jaccard score of 0.54 was obtained by the mere application of the U-Net on the SAR images dataset. To obtain an accurate segmentation of the oil spill instances, a balance between the oil spill and background classes may not be feasible. Yet, most of the successful solutions introduced by the related work [1,2,3,4,5,6], which achieved an oil spill detection accuracy of over 92%. This relied on the extraction of specific handcrafted features from SAR images such as object standard deviation, object power to mean ratio, and background standard deviation.

Recent deep-learning-based methods [9,10,11,12,13,14,15,16,17,18,19,20,21,22,23,24,25,26,27,28] that used CNN structures for both automated feature extraction, as well as classification of SAR images have relied on the use of patches to reduce the background concentration in the tested images. Pre-trained models, such as ResNet 101, VGG-16, and GAN networks as in [10,11,12,13,14,15,16,17,18,19,20,21,22,23,24,25,26,27,28] or multi-level CNN networks as in [9], were introduced to classify patches with modest performance (i.e., precision and Dice score). In [23], authors obtained improved results via introducing the use of VGG-16 and dark batch generation algorithm on spaceborne SAR images. However, we believe that the direct application of deep-learning structures may not be sufficient for accurate and sensitive predictions due to the unbalanced nature of pixel-wise classes even when pre-processing and image enhancement are adopted. This was also experimentally shown in this study, where the direct use of U-Net and SegNet models provided poor precision as well as Dice scores.

Our approach relied on patch generation from SAR images with an emphasis on creating balanced patches and reducing bias towards the background class. We have noticed that by discarding patches with very low oil spill presence using a proposed novel CNN structure, we were able to improve the outcome of the semantic segmentation framework. Training the five-stage U-Net on balanced patches with 40–60% oil spill presence and testing on patches generated by the 23-layer CNN with significant oil pixel distribution (i.e., more than 1% of the patch labelled as an oil spill) elevated the precision to 84% and *f*-1 score to 80%.

The limitations of the proposed framework are as follows: (1) The proposed two-stage deep-learning network provides an improved pixel-wise classification into oil spills and background, and it is not useful for a multi-class problem, including other targets such as ships and look-alikes. However, most of the related work, as well as our work, were interested in identifying oil spills as it is of significant importance as compared with ships and look-alikes. This will allow the removal of oil leaks and the protection of marine ecosystems. (2) Although the proposed method managed to improve the overall performance of the deep-learning-based semantic segmentation of oil spills based on SAR images, it does not consider the segmentation of patches with insignificant oil spill concentration. However, we have been able to identify the oil spill pixels and patterns with high accuracy and precision which will be potentially sufficient for dispersants, booms, and skimmers to clean up the detected oil leaks. We are also planning to address this problem in future work. (3) Although several handcrafted features have been presented in the literature that supports an accurate prediction for a neural network classifier, the use of deep-learning to detect oil spills does not offer a justification for the classification decision. Accordingly, we will use feature visualization techniques and heat maps to present the unique features based on which the model came up with an accurate prediction. (4) The use of unsupervised learning (e.g., Autoencoders) has been investigated and adopted for efficient classification of oil spill pixels. Therefore, we plan to study and investigate the application of an unsupervised Autoencoder approach that generates unique compressed codes for pixels of different classes prior to the use of a supervised semantic segmentation network such as U-Net that will potentially provide superior performance as compared with the use of either unsupervised or supervising learning.

## 6. Conclusions

In this paper, we have introduced a deep-learning framework to identify oil spill instances in SAR images. The proposed framework starts with a patch generation where each image of 1250 × 650 × 3 is split into patches of 64 × 64 × 3, followed with a frost filter to reduce the speckle noise in the background to reduce the false positive rate as oil spill as well as look-alikes appear dark in images. Filtered patches are then provided to a novel 23-layer CNN that was trained on 80% of the patches for 40 epochs and was validated via a five-fold and ten-fold cross-validation in order to screen patches with significant oil spill pixel distribution (i.e., more than 1% oil spill). The proposed model offered a superior classification performance at an accuracy, sensitivity, specificity, and weighted Kappa of almost 99%. Further, the proposed CNN stage runs the successfully classified patches with more than 1% oil spill through a robust semantic segmentation stage with a five-stage U-Net and a cost-sensitive loss function (i.e., generalized Dice loss) optimized to reduce the bias among the patches since Oil spill still may represent less than 50% of the patch resolution. The final framework was able to identify oil spill pixels with a precision of 84%, and a Dice score of 0.8 outperforming the state-of-the-art CNN introduced by Hidalgo et al. [10] and providing quite comparable performance to the latest state-of-the-art CNN introduced by Zeng et al. [23]. In addition, the proposed framework performed better on patches of size 64 × 64 as compared with 32 × 32 or 128 × 128 patches in terms of precision and Dice scores. Further, the proposed approach proved to be robust and accurate compared to the direct application of semantic segmentation models.

## Figures and Tables

**Table 1:** Classes of instances and respective labels.

Class	3-D Masks	1-D Labels	Pixel Counts
Sea	Black	0	225,071,583
Oil Spill	Cyan	1	3,188,485
Look-Alike	Red	2	13,585,532
Ship	Brown	3	87,287
Land	Green	4	9,942,113

**Table 2:** Layers of the Proposed CNN.

Layer	Kernel Size	No. of Kernels	Kernel Features	No. of Layers
Input Layer	64 × 64 × 3	1	True Color	1
Convolutional/ReLU Layers	11 × 11	32	Same Padding	4
Pooling Layer	2 × 2	32	No Padding	1
Convolutional/ReLU Layers	9 × 9	64	Same Padding	4
Pooling Layer	2 × 2	64	No Padding	1
Convolutional/ReLU Layers	7 × 7	128	Same Padding	4
Pooling Layer	2 × 2	128	No Padding	1
Fully Connected/ReLU Layers	128, 64, 32, 16	1		4
Fully Connected Layer	2	1		1
SoftMax Layer		1		1
Classification Layer		1		1

**Table 3:** First-Stage CNN Performance.

Measure	5-Fold (Mean)	5-Fold (Deviation)	10-Fold (Mean)	10-Fold (Deviation)
Training Accuracy	1	8.1 × 10^−5^	1	2.1 × 10^−4^
Validation Accuracy	0.99	1.2 × 10^−4^	0.99	2.8 × 10^−4^
Weighted Kappa Score	0.99	2.3 × 10^−4^	0.99	5.6 × 10^−4^
Sensitivity	0.99	2.2 × 10^−4^	0.99	6.1 × 10^−4^
Specificity	0.99	2.2 × 10^−4^	0.99	2.3 × 10^−4^
AUC	0.99	0.0016	0.99	0.0021

**Table 4:** Second-Stage U-Net Performance on Training Dataset.

Measure	Accuracy	Precision	Recall	Dice
**Value**	0.9	0.91	0.88	0.89

**Table 5:** Second-Stage U-Net Performance on Testing Dataset.

Measure	Accuracy	Precision	Recall	Dice
**Value**	0.92	0.84	0.76	0.8

**Table 6:** Comparison between the proposed framework and direct application of semantic segmentation.

Measure	Proposed Framework	U-Net	SegNet (VGG-19)
**Accuracy**	92%	95%	96%
**Recall**	76%	76%	66%
**Precision**	84%	27%	26%
**Dice**	80%	40%	38%

**Table 7:** Proposed framework versus variations of the proposed segmentation network.

Measure	Proposed Framework	Patch Size 32 × 32	Patch Size 128 × 128	Recall Loss Optimization	Jaccard Loss Minimization
**Accuracy**	92%	80%	94%	92%	92%
**Recall**	76%	88%	70%	76%	76%
**Precision**	84%	63%	81%	83%	84%
**Dice**	80%	74%	75%	79%	80%

**Table 8:** Comparative study between the proposed framework and the-state-of-the-art methods.

Measure	Proposed Framework	Hidalgo et al. [10]	Zeng et al. [23]
**Accuracy**	92%	99%	94%
**Recall**	76%	86.8%	83.5%
**Precision**	84%	65%	85.7%
**Dice**	80%	71%	84.6%

## Data Availability

Data sharing is not applicable to this article.

## References

[1] Calabresi G., Del Frate F., Lichtenegger J., Petrocchi A., Trivero P. Neural networks for oil spill detection using ERS-SAR data. Proceedings of the IEEE 1999 International Geoscience and Remote Sensing Symposium. IGARSS’99 (Cat. No.99CH36293).

[2] De Souza D.L., Neto A.D.D., da Mata W. Intelligent system for feature extraction of oil slick in SAR images: Speckle filter analysis. Proceedings of the International Conference on Neural Information Processing.

[3] Stathakis D., Topouzelis K., Karathanassi V. Large-scale feature selection using evolved neural networks. Proceedings of the Image and Signal Processing for Remote Sensing XII.

[4] Topouzelis K., Karathanassi V., Pavlakis P., Rokos D. (2007). Detection and discrimination between oil spills and look-alike phenomena through neural networks. ISPRS J. Photogramm. Remote Sens..

[5] Singha S., Bellerby T.J., Trieschmann O. (2013). Satellite Oil Spill Detection Using Artificial Neural Networks. IEEE J. Sel. Top. Appl. Earth Obs. Remote Sens..

[6] Song D., Ding Y., Li X., Zhang B., Xu M. (2017). Ocean Oil Spill Classification with RADARSAT-2 SAR Based on an Optimized Wavelet Neural Network. Remote Sens..

[7] Chen G., Li Y., Sun G., Zhang Y. (2017). Application of Deep Networks to Oil Spill Detection Using Polarimetric Synthetic Aperture Radar Images. Appl. Sci..

[8] Gallego A.-J., Gil P., Pertusa A., Fisher R.B. (2019). Semantic Segmentation of SLAR Imagery with Convolutional LSTM Selectional AutoEncoders. Remote Sens..

[9] Orfanidis G., Ioannidis K., Avgerinakis K., Vrochidis S., Kompatsiaris I. A deep neural network for oil spill semantic segmentation in Sar images. Proceedings of the 25th IEEE International Conference on Image Processing (ICIP).

[10] Nieto-Hidalgo M., Gallego A.-J., Gil P., Pertusa A. (2018). Two-Stage Convolutional Neural Network for Ship and Spill Detection Using SLAR Images. IEEE Trans. Geosci. Remote Sens..

[11] Yu X., Zhang H., Luo C., Qi H., Ren P. (2018). Oil Spill Segmentation via Adversarial *f*-Divergence Learning. IEEE Trans. Geosci. Remote Sens..

[12] Yin L., Zhang M., Zhang Y., Qiao F. (2018). The long-term prediction of the oil-contaminated water from the Sanchi collision in the East China Sea. Acta Oceanol. Sin..

[13] Gallego A.-J., Gil P., Pertusa A., Fisher R.B. (2018). Segmentation of Oil Spills on Side-Looking Airborne Radar Imagery with Autoencoders. Sensors.

[14] Guo H., Wei G., An J. (2018). Dark Spot Detection in SAR Images of Oil Spill Using Segnet. Appl. Sci..

[15] Li Y., Zhang Y., Yuan Z., Guo H., Pan H., Guo J. (2018). Marine Oil Spill Detection Based on the Comprehensive Use of Polarimetric SAR Data. Sustainability.

[16] Jiao Z., Jia G., Cai Y. (2019). A new approach to oil spill detection that combines deep learning with unmanned aerial vehicles. Comput. Ind. Eng..

[17] Zhu X., Li Y., Zhang Q., Liu B. (2019). Oil Film Classification Using Deep Learning-Based Hyperspectral Remote Sensing Technology. ISPRS Int. J. Geo-Inf..

[18] Krestenitis M., Orfanidis G., Ioannidis K., Avgerinakis K., Vrochidis S., Kompatsiaris I. (2019). Oil Spill Identification from Satellite Images Using Deep Neural Networks. Remote Sens..

[19] Qiao F., Wang G., Yin L., Zeng K., Zhang Y., Zhang M., Xiao B., Jiang S., Chen H., Chen G. (2019). Modelling oil trajectories and potentially contaminated areas from the Sanchi oil spill. Sci. Total. Environ..

[20] Yang J.-F., Wan J.-H., Ma Y., Zhang J., Hu Y.-B., Jiang Z.-C. (2019). Oil Spill Hyperspectral Remote Sensing Detection Based on DCNN with Multi-Scale Features. J. Coast. Res..

[21] Park S.-H., Jung H.-S., Lee M.-J., Lee W.-J., Choi M.-J. (2019). Oil Spill Detection from PlanetScope Satellite Image: Application to Oil Spill Accident near Ras Al Zour Area, Kuwait in August 2017. J. Coast. Res..

[22] Liu B., Li Y., Li G., Liu A. (2019). A Spectral Feature Based Convolutional Neural Network for Classification of Sea Surface Oil Spill. ISPRS Int. J. Geo-Inf..

[23] Zeng K., Wang Y. (2020). A Deep Convolutional Neural Network for Oil Spill Detection from Spaceborne SAR Images. Remote. Sens..

[24] Yekeen S.T., Balogun A., Yusof K.B.W. (2020). A novel deep learning instance segmentation model for automated marine oil spill detection. ISPRS J. Photogramm. Remote Sens..

[25] Yekeen S.T., Balogun A.L. (2020). Automated Marine Oil Spill Detection Using Deep Learning Instance Segmentation Model. Int. Arch. Photogramm. Remote Sens. Spatial Inf. Sci..

[26] Bianchi F., Espeseth M., Borch N. (2020). Large-Scale Detection and Categorization of Oil Spills from SAR Images with Deep Learning. Remote Sens..

[27] Zhang J., Feng H., Luo Q., Li Y., Wei J., Li J. (2020). Oil Spill Detection in Quad-Polarimetric SAR Images Using an Advanced Convolutional Neural Network Based on SuperPixel Model. Remote Sens..

[28] Baek W., Jung H., Kim D. (2020). Oil spill detection of Kerch strait in November 2007 from dual-polarized TerraSAR-X image using artificial and convolutional neural network regression models. J. Coast. Res..

[29] Copernius Open Access Hub. https://scihub.copernicus.eu/.

[30] Oil Spill Detection Dataset. https://mklab.iti.gr/results/oil-spill-detection-dataset/.

[31] Shi Z., Fung K. A comparison of digital speckle filters. Proceedings of the IGARSS ’94—1994 IEEE International Geoscience and Remote Sensing Symposium.

[32] Ronneberger O., Fischer P., Brox T. (2015). U-Net: Convolutional Networks for Biomedical Image Segmentation. Proceedings of the International Conference on Medical Image Computing and Computer-Assisted Intervention.

[33] Sudre C.H., Li W., Vercauteren T., Ourselin S., Cardoso M.J. (2017). Generalised Dice Overlap as a Deep Learning Loss Function for Highly Unbalanced Segmentations. Deep Learning in Medical Image Analysis and Multimodal Learning for Clinical Decision Support.

[34] Badrinarayanan V., Kendall A., Cipolla R. (2017). SegNet: A Deep Convolutional Encoder-Decoder Architecture for Image Segmentation. IEEE Trans. Pattern Anal. Mach. Intell..

